# Nature of the Lithium Amide–Imide Catalyst System under Ammonia Decomposition Conditions

**DOI:** 10.1021/acs.jpcc.5c04123

**Published:** 2025-10-03

**Authors:** Thomas J. Wood, Eleanor G. Frew

**Affiliations:** † ISIS Pulsed Neutron and Muon Source, Rutherford Appleton Laboratory, Harwell Oxford, Didcot OX11 0QX, U.K.; ‡ Inorganic Chemistry Laboratory, 6396 University of Oxford, Oxford OX1 3QR, U.K.

## Abstract

The varying stoichiometry of the lithium amide–imide ammonia decomposition catalyst under working conditions was ascertained by calculating the gas release events in the ammonia decomposition experiments. Rather than varying smoothly between amide and imide, the gas releases showed evidence of a molten state with a majority of amide stoichiometry before converting at temperatures above 460 °C to a solid with a majority of imide stoichiometry. At higher temperatures, there was indirect evidence of nitride hydride groups being formed within the catalyst structure.

## Graphical abstract

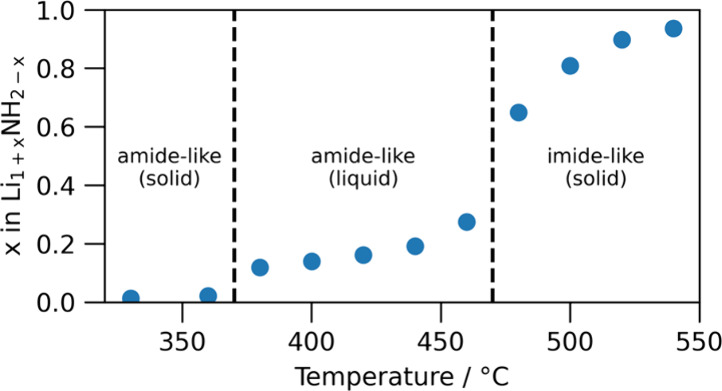

## Introduction

Greenhouse gas emission and the resultant climate change remain some of the most urgent scientific and technological challenges of this century.^1^ As the cost of renewable power sources, such as solar or wind, falls and, therefore, market penetration increases, issues of intermittency and, thus, energy storage/transport become ever more important.^2^ While secondary batteries (especially lithium-ion) are able to mitigate short-term fluctuations in power usage, they are not a viable solution for longer-term (e.g., interseasonal) storage or, indeed, for transporting energy from, for example, solar-rich areas to more densely populated regions where the energy demand is higher.^3^ For these applications, and others where energy density is paramount, chemical energy storage is likely to be the most practical solution, and ammonia is one of the few carbon-free prospects. It has the advantages of there already being massive international infrastructure in place (thanks to the fertilizer industry) and an energy density of 2.9 kWh L^–1^ LHV (greater than liquid hydrogen).^3,4^

Although ammonia may be used as an energy storage medium, it may also be used for transporting and storing hydrogen required as a green-reducing agent in industrial processes (e.g., steelmaking).^5,6^ Both energy storage and chemical reductant applications usually entail the full or partial decomposition of ammonia into its elemental constituents (hydrogen and nitrogen). Current transition metal ammonia decomposition catalysts partially mirror the most active ammonia synthesis catalysts: Ruthenium shows the greatest activity followed by nickel and iron (dependent on conditions).^7–10^ Recently, however, a new class of catalysts based on light metal amides and imides has been uncovered, which operates under a different mechanism to the transition metal catalysts.^11–13^ To date, the most effective light metal catalyst has been the lithium amide–imide system (with or without transition metal nitrides).^12–14^ Lithium amide and lithium imide may interconvert with the release or absorption of ammonia according to eq 1:

$$2{\mathrm{LiNH}}_{2}⇌{\mathrm{Li}}_{2}\mathrm{NH}+{\mathrm{NH}}_{3}$$ 1

Lithium imide adopts a cubic structure with a face-centered cubic array of nitrogen atoms and lithium atoms in all the tetrahedral holes (*Fm*3̅*m* space group) at temperatures above 90 °C.^15^ The lithium amide structure also features a face-centered array of nitrogen atoms but with lithium occupying only half the tetrahedral holes, making a tetragonal structure (*I*4̅ space group).^16^ Previous studies on this system, in the context of its potential use for reversible hydrogen storage, have shown that the lithium amide and lithium imide will form a solid solution Li_1+*x*_NH_2–*x*_ for much of the range 0 < *x* < 1.^17,18^ A recent, high resolution, X-ray diffraction study showed that there are, in fact, two closely related amide structures, differing only by the ordering of the lithium atoms, one structure adopting the *I*4̅ space group and the other the *P*4̅ space group.^19^ These amide structures persisted up to *x* values between 0.25 and 0.33, after which the disordered *Fm*3̅*m* imide structure was able to accommodate the amide species. Importantly, for a practical catalyst system, when all the amide groups were able to be adopted within the *Fm*3̅*m* structure, the system was solid until decomposition; for lower values of *x*, the amide–imide melted at around 370 °C.

The structure of lithium amide–imide has been well characterized ex situ, as outlined above, but the only in situ analyses of its structure under ammonia decomposition conditions remain in situ neutron diffraction,^13^ where liquid or amorphous phases are difficult to characterize. In this study, residual gas analysis via mass spectrometry and flow measurements is used to quantify both the decomposition activity of the catalyst at various temperatures and gas releases at each temperature step, which, in turn, give an approximate value for the stoichiometry of the catalyst at each stage.

## Experimental Section

Ammonia decomposition experiments were performed in a cylindrical stainless steel (316L) reactor consisting of a tube within a tube (9 mm diameter). Lithium amide (0.4 g, 95%, Acros Organics), used as supplied, was loaded into the reactor in a glove box under an inert atmosphere (argon, <0.1 ppm oxygen, and <0.1 ppm water); the catalyst was located such that gas flowed over rather than through the sample. The reason for the flow-over setup was to mitigate any problems associated with a change of state of the catalyst, since it was expected to melt under certain conditions during the experiment (the flow-through setup would likely lead to the blocking of the reactor or excessive catalyst escape). The reactor was coupled to a gas flow rig consisting of two flow controller inlets (Teledyne Hastings, HFC302, joined by T-piece to the reactor), a baratron to measure pressure, and a flow meter (Teledyne Hastings, HFM300) on the outlet; the reactor itself was placed inside a tube furnace (Severn Thermal Solutions), and the temperature of the reactor was monitored by a *K*-type thermocouple placed inside a sleeve and inserted into the reactor (Figure 1). The flow meter and controllers had previously been calibrated to ammonia. A mass spectrometer (Hiden Analytical HPR-20 QIC R&D) in histogram mode (MASsoft 7) monitored the gas species exiting the reactor between *m*/*z* = 1 and 40 (inclusive) and was connected via a T-piece with an exhaust tube, such that the system after the flow controllers was running at close to ambient pressure (1 bar). Prior to the start of the experiment, the rig up to the reactor was placed under vacuum to remove any air, and subsequently ammonia (99.98%, refrigerant grade, BOC) was introduced at a flow rate of 30 sccm (standard cubic centimeters per minute); this was invariant throughout the experiments. A period of 5 min was allowed before starting the temperature program in order to make sure any argon in the reactor had been flushed out of the rig by the flowing ammonia; this was confirmed by viewing the mass spectrometry output. The furnace was controlled by a bespoke LabView program and was ramped at a rate of 5 °C min^–1^ to 300 °C, with a dwell of 4 h; after that, the following temperatures were used: 330, 360, 380, 400, 420, 440, 460, 480, 500, 520, and 540 °C, all with dwells of 2 h. The furnace was then cooled at the same rate of 5 °C min^–1^ to room temperature before the rig was flushed through with argon gas. The remaining catalyst was then unloaded under inert gas conditions in the glove box.

**Figure 1:**
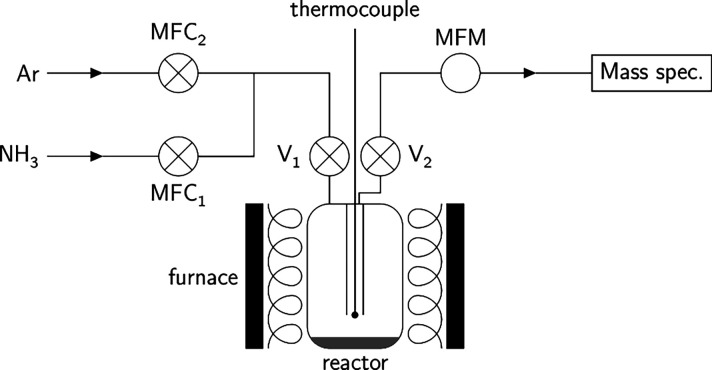
Diagram of the experimental setup used; the catalyst was located at the bottom of the reactor.

Prior to conducting ammonia decomposition experiments, the mass spectrometer was calibrated with known mixtures of argon, ammonia, nitrogen, and hydrogen to determine the fragmentation ratios of each of those gases (i.e., the fingerprint patterns in the *m*/*z* histograms) and also the ionization factor of each species (relative to argon, which was assigned an ionization factor of 1). A bespoke Python program was used to establish the fraction of each gas in the outflow at each time step from these values. Where possible, each experiment was internally calibrated as a check in order to minimize any effect of drift in the hydrogen ionization factor.

In order to extract gas component flows (i.e., the flow of each individual gas species), the mass spectrometry data were combined with the flow data. In order to do this, the time delay, *t*_d_, between the reactor and the mass spectrometer has to be taken into account (the mass flow meter essentially measures the instantaneous flow out of the reactor). This time delay is a function of the volume of the pipework (which remains fixed at around 100 cm^3^) and the flow. The flow measurement of the mass flow meter, however, is dependent on the gas species flowing through it; each gas species has its own gas conversion factor, as supplied by the manufacturer. This is straightforward to calculate while the system is in equilibrium because at that point, *t*_d_ will be constant as will the mass spectrometry output. Under dynamic conditions (e.g., changing temperature), however, in this study, the time delay at each time point, *t*_*i*_, was calculated by evaluating the average measured flow in the range [*t*_*i*_, *t*_*i*_ + *t*_d_] and the average gas fractions in the range [*t*_*i*_ + *t*_d_, *t*_*i*_ + 2*t*_d_]. From these values, the actual flow (accounting for gas conversion factors) could be calculated, and, therefore, the value for *t*_d_ could be updated. One iteration per time point was found to be sufficient to accurately converge these values for the conditions in this study. The individual gas component flows were extracted by combining the gas fraction values at *t*_*i*_ + *t*_d_ with the actual flow at *t*_*i*_. The iterative process was initialized from points where the reaction was clearly in equilibrium at the end of temperature dwells (several points for each experiment were taken in order to verify the accuracy of this method).

As the ammonia decomposition reaction proceeds, the expected flow out, *F*_exp_, is a function of the extent of the reaction (conversion), α, and the flow in, *F*_in_:

$$F_{\exp}=(1+\alpha )F_{\mathrm{in}}$$ 2

This is because two moles of gas are produced from every one mole of ammonia that decomposes. This expected flow was calculated from the gas fractions at each point to provide a background function. Any significant deviation from this background function was treated as either a gas release or an absorption event.

X-ray diffraction patterns were taken using an X’Pert machine (Panalytical) equipped with a copper Kα source over the two-theta range 10–60°. Samples were loaded in an argon-filled glove box onto a flat-plate configuration air-sensitive sample holder.

## Results and Discussion

Lithium amide–imide is well-known as a catalyst system for ammonia decomposition, showing greater activity than even the state-of-the-art ruthenium.^13^ Characterization of its catalytic ability is typically shown in the form of a sigmoid curve showing the ammonia conversion fraction versus temperature under a constant flow of ammonia once equilibrium has been achieved. In Figure 2, sigmoid curves of this type can be seen for lithium amide and for the empty (“blank”) reactor. The empty reactor shows some ammonia decomposition over 400 °C because it is made of stainless steel, which is comprised, in large part, of iron and nickel, both ammonia decomposition catalysts in their own right. Ideally, an inert reactor would be used for these experiments: For example, a standard reactor material for many catalytic reaction studies is quartz. For lithium amide–imide, however, this is not possible since it is strongly basic and has been shown to react with a variety of oxides under ammonia decomposition reaction conditions including silica and alumina.^20^ Under the temperature regime in this study (up to 540 °C), however, the blank reactor only reaches conversion fractions of <0.6, whereas the lithium amide–imide catalyst reaches nearly complete conversion. The data in Figure 2 have been fitted to a Gompertz function, which can be derived from assuming that the ammonia decomposition reaction follows a single mechanism, which is first order with respect to the partial pressure of ammonia. This does a good job of fitting the lithium amide curve; however, there is an anomaly at 480 °C, which is significantly outside the uncertainties of the measurement (±2%). Another issue with data presented in the sigmoid form is that the behavior of the catalyst under nonequilibrium conditions (i.e., immediately upon being heated or cooled) is lost. For most catalytic systems, where the stoichiometric nature of the catalyst is invariant over the temperature range of interest, this nonequilibrium behavior is uninformative; however, for the lithium amide–imide system, it is already known that the lithium amide–imide exists as a solid solution, Li_1+*x*_NH_2–*x*_, where the value of *x* will depend on the temperature and the ratio of ammonia/nitrogen/hydrogen partial pressures.^13^

**Figure 2:**
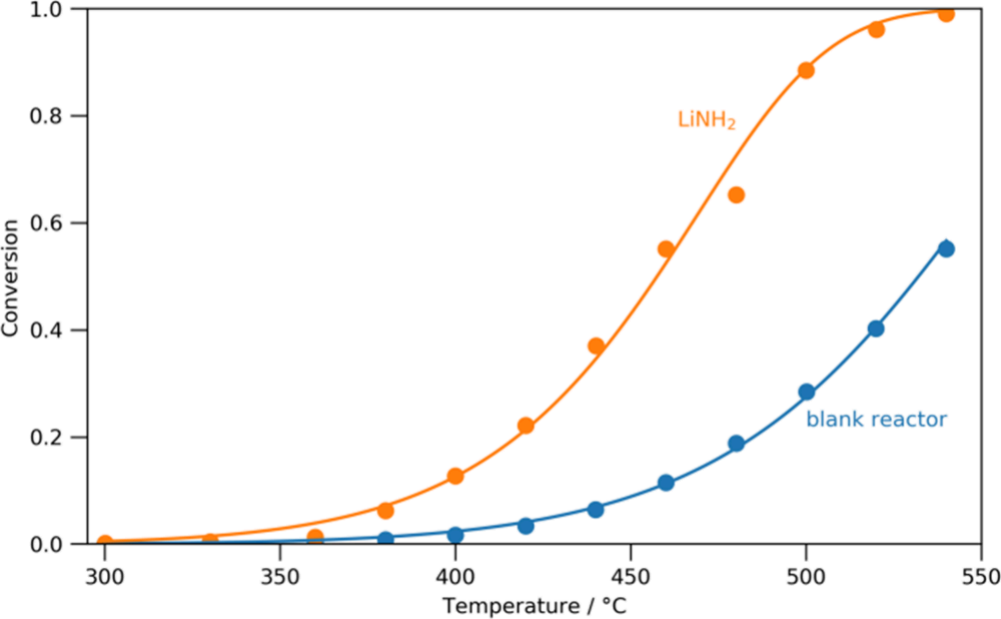
Sigmoids of the ammonia conversion versus temperature for the empty (“blank”) reactor and lithium amide catalyst without any removal of the blank reactor contribution (i.e., lithium amide + “blank”).

In the experimental setup used in this study, a mass flow meter and a mass spectrometer were used to monitor the outflow and composition of the gases from the reactor (Figure 1). Marrying these two sets of data enables the extraction of individual component gas flows for ammonia, nitrogen, and hydrogen. These component gas flows for the blank reactor are shown in Figure S1. As the temperature is stepped up, the total flow increases, which is expected from the fact that one mole of ammonia decomposes into a total of two moles of hydrogen and nitrogen (in a 3:1 ratio). The gray lines on the gas flow traces are the expected gas component flows if the only contributions to the outflow are unreacted ammonia or the nitrogen and hydrogen products of ammonia that has decomposed (i.e., *F*_exp_ from eq 2); these expected flows can be straightforwardly calculated from the known flow of ammonia in and the partial pressures of the gases. As expected for the blank reactor, the vast majority of the contribution to the flow is from these two sources (unreacted and decomposed ammonia); however, it is just possible to see a small increase in the total flow at some temperatures immediately upon heating. These flow increases are short-lived. In contrast, the gas component flows for lithium amide–imide show much larger flow increases at every temperature (Figure 3), with quite marked deviations from the expected flow from eq 2. These flow increases are associated with gas release, either from the walls of the reactor or from the catalyst itself.

**Figure 3:**
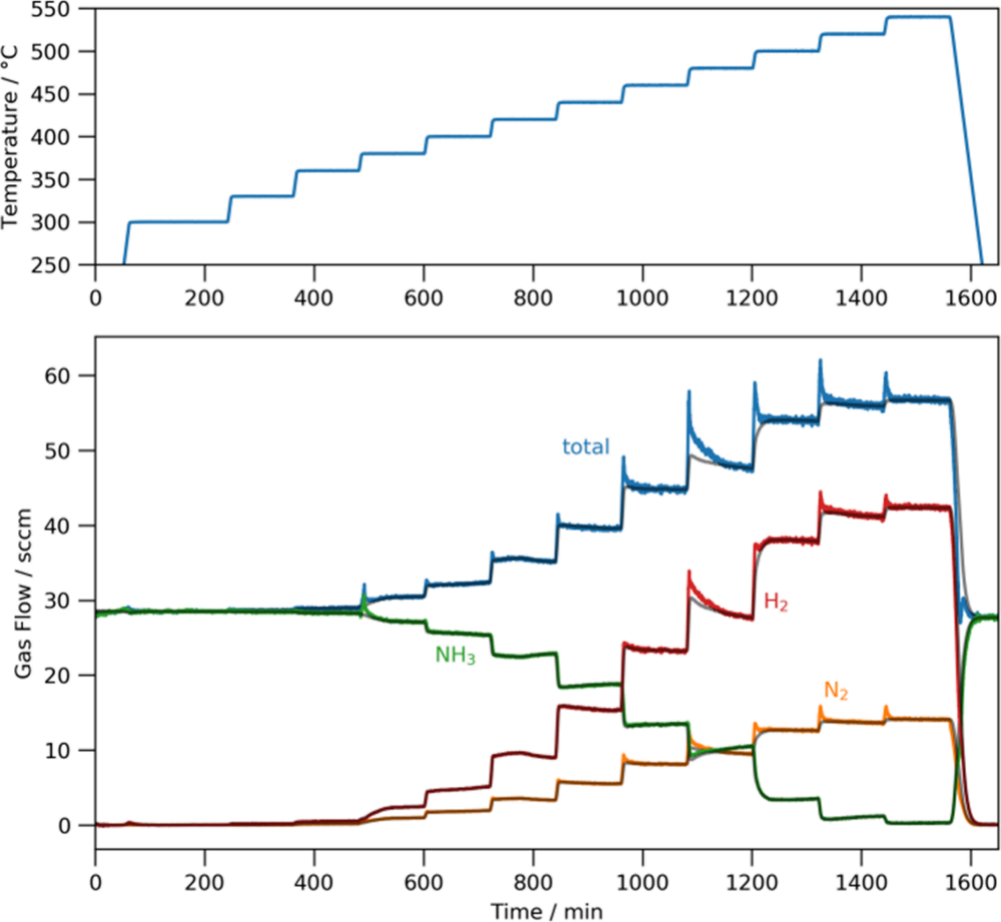
Gas component flows and temperature profile for the lithium amide–imide system; gray lines denote the expected flow from a system where only the unreacted or decomposed ammonia comprises the outflow.

Figure S2 and Figure 4 show the difference between the gas component flows and the expected flow from a system with no gas desorption/absorption from the reactor or catalyst (behaving according to eq 2); the figures are plotted on the same scale. The gas releases from the blank reactor are very small and comprised almost exclusively of ammonia up to 480 °C (∼1100 min) and mixtures of ammonia, nitrogen, and hydrogen thereafter. These small releases can be explained by the release of adsorbed ammonia on the surface of the reactor; at higher temperatures, some of the ammonia thus released will decompose into hydrogen and nitrogen before exiting the reactor. The reabsorption of ammonia can be seen upon cooling. There is slightly more nitrogen released than expected from just ammonia desorption, but this is likely to be due to small amounts of iron denitriding, which is known to occur under these conditions.^21,22^ By contrast, there are large amounts of ammonia gas released from much lower temperatures with the lithium amide. The initial release of ammonia at 380 °C coincides with the melting point of lithium amide and is consistent with previous simultaneous thermal analysis (differential scanning calorimetry combined with thermogravimetric analysis) results.^13^ After this initial release of ammonia, there are further smaller releases of ammonia at each temperature combined with small amounts of nitrogen and hydrogen until 480 °C, whereupon much larger amounts of ammonia, nitrogen, and hydrogen are released. The largest release of gas occurs at precisely the anomalous temperature seen in the sigmoid. These releases then decrease in magnitude until by the final temperature (540 °C), almost equimolar amounts of nitrogen and hydrogen are released. Given the very small volumes of gas released by the blank reactor, it is reasonable to infer that the vast majority of these gas releases is from the catalyst and specifically as it changes to a more imide-like stoichiometry (i.e., as *x* increases in Li_1+*x*_NH_2–*x*_). Large quantities of nitrogen, hydrogen, and ammonia are reabsorbed on cooling, which is consistent with the reformation of lithium amide.

**Figure 4:**
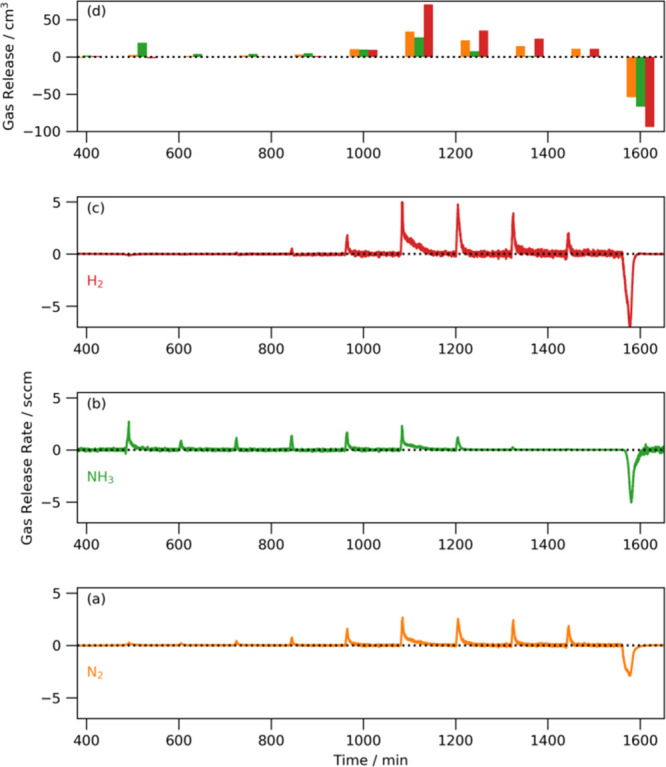
Flow due to gas released from the walls of the reactor and the lithium amide catalyst for (a) nitrogen, (b) ammonia, (c) hydrogen, and (d) total volumes of gases released per temperature (bars offset from each other for clarity; orange = nitrogen, green = ammonia, red = hydrogen).

Values for the stoichiometry of the lithium amide catalyst (*x* in Li_1+*x*_NH_2–*x*_) can be calculated from the gas released at each temperature, and these are shown in Figure 5 along with the excess gas released. This excess gas refers to the gas left over after ammonia, and a 3:1 ratio of hydrogen to nitrogen has been used to calculate the change in *x*. In Figure 5a, it can be seen that this excess gas is exclusively composed of nitrogen. This is probably partially due to the increased denitriding of the stainless steel reactor from the blank reactor case, which would be expected given the higher overall hydrogen partial pressure at a given temperature when the amide catalyst is present. The overall values of the excess nitrogen gas released are relatively small. Considering the change in the stoichiometry of the lithium amide catalyst, it is clear that there is an initial shift to a more imide-like stoichiometry at 380 °C (*x* changes to 0.12) just above the melting point of the amide, but until 480 °C, there are only very small shifts in the stoichiometry; *x* ≈ 0.27 at 460 °C. A recent study of a series of lithium amide–imide samples over the range of *x* values concluded that for *x* ≤ 0.25, the solid solution will take on some of the amide-like tetragonal structure.^19^ These materials all melted at temperatures of around 370 °C and, therefore, would be molten under the conditions experienced in this study. At 480 °C, however, the value of *x* leaps to 0.65 with a (relatively) large amount of concomitant gas release. This indicates that the catalyst has now taken on the disordered imide structure (*Fm*3̅*m*, face-centered cubic array of nitrogens, lithiums occupying all the tetrahedral sites, and hydrogens disordered), as has been previously seen in in situ neutron diffraction studies on this system.^13^ Significantly, solid solutions in this range have no melting point;^19^ instead, they decompose to lithium nitride hydride (along with release of equimolar amounts of nitrogen and hydrogen) via:

$$2{\mathrm{Li}}_{2}{\mathrm{NH}}_{\left(s\right)}\to {\mathrm{Li}}_{4}{\mathrm{NH}}_{\left(s\right)}+1/2N_{2\left(g\right)}+1/2H_{2\left(g\right)}$$ 3

**Figure 5:**
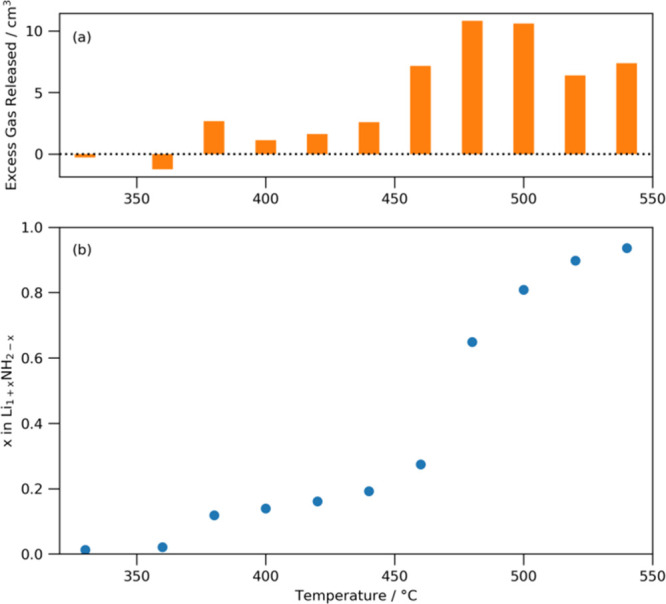
(a) Excess nitrogen gas released per temperature that cannot be accounted for by the catalyst stoichiometry shift from amide to imide; (b) stoichiometry of the catalyst.

This likely change of state of the catalyst from a molten amide-like structure to a solid imide-like structure explains the depression of the 480 °C point in the sigmoid in Figure 2, since the solidification of the catalyst will probably lead to less surface area, with which the gases interact. There may also be a beneficial effect of imide and amide ions being able to freely move within the molten salt, which is lost upon solidification. Between 480 and 540 °C, the value of *x* increases until it reaches 0.94, i.e., almost entirely imide in nature.

The values of *x* shown in Figure 5, in fact, represent the maximum values of *x* if and only if (i) all the ammonia released is due to amide to imide conversion and (ii) all the hydrogen released is due to ammonia released by amide to imide conversion, which subsequently decomposes. Lithium nitride hydride is known to form a solid solution itself with lithium imide,^19^ so it is likely that the equimolar release of nitrogen and hydrogen at 540 °C (see Figure 4d) in this study is from the formation of nitride hydride groups from imide. There has previously been circumstantial evidence for lithium nitride hydride (or, at least, lithium-rich intermediates) occurring during ammonia decomposition; this was obtained via isotopic labeling studies over lithium amide–imide catalysts.^23^ It is likely, therefore, that the lower temperature nitrogen releases are dominated by reactor wall denitriding (as in the blank reactor case), but at higher temperatures (≥480 °C), this excess nitrogen is, in fact, due to the formation of some nitride hydride groups within the catalyst. Indeed, the ability of the *Fm*3̅*m* imide structure to accommodate substantial fractions of nitride hydride groups (in lieu of imide) has been shown by X-ray diffraction studies.^19^

Figure 6 shows close ups of gas release upon likely lithium amide melting and gas reabsorption upon cooling. The ammonia release between 360 and 380 °C shows some complexity (Figure 6a). First, there is a small absorption of ammonia, which is explained by the lithium amide catalyst being not quite stoichiometric (this has been seen previously in high resolution X-ray diffraction measurements^19^). There is then an initial release of ammonia between 485 and 489 min (up to 1.0 sccm), which occurs over the temperature range 375–380 °C. This initial release is then followed by two further releases (the peaks at 491 and 492 min) once the temperature has reached 380 °C. The most plausible explanation is that the lithium amide exists as a solid solution range, even as *x* is close to zero. Lithium amide–imide samples with *x* ≤ 0.25 have been shown to exhibit a range of melting points over the range 360–380 °C, where there is a slight negative correlation between *x* and the melting point value.^19^ This is consistent with the data presented in this study, where release of ammonia and subsequent increase in *x* precipitates further melting of the sample.

**Figure 6:**
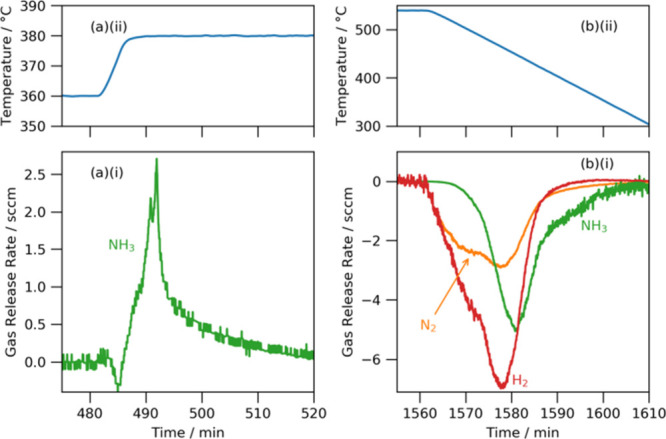
(a) (i) Ammonia release rate at temperatures between 360 and 380 °C (nitrogen and hydrogen traces have been removed for clarity, since they show only small deviations from zero) and (ii) temperature trace of the same region; (b) (i) ammonia, nitrogen, and hydrogen release rates upon cooling and (ii) temperature trace of the same region for the lithium amide catalyst.

The shapes of the gas absorption traces upon cooling show further evidence for the formation of some nitride hydride groups at higher temperatures and higher conversions (Figure 6b). In the initial part of cooling, the sample reabsorbs equimolar amounts of nitrogen and hydrogen, which would be expected from the reaction of ammonia with lithium nitride hydride to form lithium imide (at these temperatures, almost all the ammonia has decomposed to nitrogen and hydrogen; hence, why there is no ammonia absorption peak initially). The hydrogen absorption trace then separates from the nitrogen absorption trace at temperatures below 535 °C, which indicates a mixture of nitride hydride to imide conversion and some imide to amide conversion (a decrease in *x*). This reabsorption by the catalyst, however, shows a significant acceleration between temperatures of 485 and 465 °C, where *x* decreases more rapidly. This is the reverse process of what was observed on heating the catalyst stepwise, where there was a large increase in *x* between 460 and 480 °C. It seems likely, therefore, that the catalyst prefers to either exist as a molten state as majority amide or in the solid state as majority imide; mixtures between 0.25 and 0.65 are fleetingly stable under the conditions experienced here. The gas release rate for both the heating and cooling can be seen in Figure S3, where, despite the cooling regime operating without dwell periods, the absorption profiles can clearly be seen to mirror the release profiles.

The value of *x* once the system has reached room temperature was calculated to be 0.27, which indicates that some of the catalysts have either escaped the system (previously an issue with light metal amide catalysts^24^) or that the catalyst retains some imide character. An X-ray diffraction pattern of the catalyst afterward showed a range of amide–imide stoichiometries, as evidenced by both amide-like tetragonal *I*4̅ and *P*4̅ structures and imide-like *Fm*3̅*m* structures visible within the pattern (Figure S4), which is consistent with this result.

## Conclusions

The ammonia decomposition catalyst lithium amide–imide has been shown to exist as a range of stoichiometries from fully amide to majority imide. The catalyst changes phase from molten (380–480 °C) when the ratio of amide to imide is less than or equal to 0.27. After this temperature, however, the catalyst goes to the majority imide and solidifies, which manifests itself as a discontinuity in the value of *x*. This has practical implications for the use of these catalysts. In general, solid catalysts are much more straightforward to deal with and raise a wealth of possibilities to increase surface area and, therefore, catalytic reaction rate. This, coupled with the fact that molten lithium amide is associated with catalyst loss,^24^ implies that the catalyst should be used only at temperatures above 480 °C if the solid nature of the catalyst is to be retained. It should be noted that these temperatures are dependent on the exact reactor setup and space velocity.

At higher temperatures, the excess nitrogen released is indicative of the formation of nitride hydride groups within the catalyst structure. This process was shown to be reversible and is consistent with the previously postulated mechanism of lithium imide and lithium nitride hydride being the active catalytic cycle.

## Supplementary Material



## Notes

Control (blank) corollaries of Figures 3 and 4; gas release and absorption rates for the catalyst plotted versus reactor temperature; powder X-ray diffraction pattern for the postexperiment catalyst (PDF)

## References

[1] Masson-Delmotte, V. ; Zhai, P. ; Pörtner, H.-O. ; Roberts, D. ; Skea, J. ; Shukla, P. R. ; Pirani, A. ; Moufouma-Okia, W. ; Péan, C. ; Pidcock, R. ; Connors, S. ; Matthews, J. B. R. ; Chen, Y. ; Zhou, X. ; Gomis, M. I. ; Lonnoy, E. ; Maycock, T. ; Tignor, M. ; Waterfield, T. Global Warming of 1.5 °C. An IPCC Special Report on the impacts of global warming of 1.5°C above pre-industrial levels and related global greenhouse gas emission pathways, in the context of strengthening the global response to the threat of climate change, sustainable development, and efforts to eradicate poverty; World Meteorological Organisation: Geneva, 2018.

[2] Gummer, J. S. ; King, J. E. ; Chater, N. ; Heaton, R. ; Johnson, P. ; Le Quéré, C. ; Skea, J. F. Hydrogen in a low-carbon economy; Committee on Climate Change: UK, 2018.

[3] Giddey S., Badwal S. P. S., Munnings C., Dolan M. (2017). Ammonia as
a Renewable
Energy Transportation Media. ACS Sustainable
Chem. Eng..

[4] David, W. I. F. ; Armstrong, F. ; Bowen, P. ; Fowler, D. ; Irvine, J. ; Torrente Murciano, L. ; Baker, D. ; Brown, T. ; Cook, N. ; Cowley, S. ; Ammonia: zero-carbon fertilizer, fuel and energy store; Royal Society: London, 2020

[5] Philibert, C. Hydrogen and Ammonia: Building Global Momentum. Presented at the Ammonia Energy Conference, Orlando, November, 2019.

[6] Lamb K. E., Dolan M. D., Kennedy D. F. (2019). Ammonia for hydrogen storage; A review
of catalytic ammonia decomposition and hydrogen separation and purification. Int. J. Hydrogen Energy.

[7] Yin S. F., Xu B. Q., Zhou X. P., Au C. T. (2004). A mini-review on
ammonia decomposition catalysts for on-site generation of hydrogen
for fuel cell applications. Appl. Catal., A.

[8] Boisen A., Dahl S., Nørskov J., Christensen C. (2005). Why the optimal
ammonia synthesis catalyst is not the optimal ammonia decomposition
catalyst. J. Catal..

[9] Hansgen D. A., Vlachos D. G., Chen J. G. (2010). Using first principles
to predict
bimetallic catalysts for the ammonia decomposition reaction. Nat. Chem..

[10] Schüth F., Palkovits R., Schlögl R., Su D. S. (2012). Ammonia as a possible
element in an energy infrastructure: catalysts for ammonia decomposition. Energy Environ. Sci..

[11] David W. I. F., Makepeace J. W., Callear S. K., Hunter H. M. A., Taylor J. D., Wood T. J., Jones M. O. (2014). Hydrogen production from ammonia
using sodium amide. J. Am. Chem. Soc..

[12] Guo J., Wang P., Wu G., Wu A., Hu D., Xiong Z., Wang J., Yu P., Chang F., Chen Z. (2015). Lithium imide synergy
with 3d transition-metal nitrides
leading to unprecedented catalytic activities for ammonia decomposition. Angew. Chem., Int. Ed..

[13] Makepeace J. W., Wood T. J., Hunter H. M. A., Jones M. O., David W. I. F. (2015). Ammonia
decomposition catalysis using non-stoichiometric lithium imide. Chem. Sci..

[14] Brooker-Davis C., Makepeace J., Wood T. (2023). Enhancement of the Catalytic Activity
of Lithium Amide towards Ammonia Decomposition by Addition of Transition
Metals. J. Ammonia Energy.

[15] Balogh M. P., Jones C. Y., Herbst J. F., Hector L. G., Kundrat M. (2006). Crystal structures and phase transformation of deuterated
lithium imide, Li2ND. J. Alloys Compd..

[16] Jacobs H., Juza R. (1972). Neubestimmung der kristallstruktur
des lithiumamids. Z. Anorg. Allg. Chem..

[17] David W. I. F., Jones M. O., Gregory D. H., Jewell C. M., Johnson S. R., Walton A., Edwards P. P. (2007). A mechanism
for non-stoichiometry
in the lithium amide/lithium imide hydrogen storage reaction. J. Am. Chem. Soc..

[18] Makepeace J. W., David W. I. F. (2017). Structural insights into the lithium amide-imide solid
solution. J. Phys. Chem. C.

[19] Makepeace J. W., Brittain J. M., Sukhwani
Manghnani A., Murray C. A., Wood T. J., David W. I. F. (2021). Compositional
flexibility in Li-N-H materials: implications
for ammonia catalysis and hydrogen storage. Phys. Chem. Chem. Phys..

[20] Wood T. J., Makepeace J. W. (2018). Assessing Potential Supports for Lithium Amide-imide
Ammonia Decomposition Catalysts. ACS Appl. Energy
Mater..

[21] Feyen M., Weidenthaler C., Güttel R., Schlichte K., Holle U., Lu A., Schüth F. (2011). High-temperature
stable, iron-based core-shell catalysts for ammonia decomposition. Chem. – Eur. J..

[22] Wood T. J., Makepeace J. W., David W. I. F. (2017). Neutron diffraction and gravimetric
study of the iron nitriding reaction under ammonia decomposition conditions. Phys. Chem. Chem. Phys..

[23] Wood T. J., Makepeace J. W., David W. I. F. (2017). Isotopic studies of the ammonia decomposition
reaction using lithium imide catalyst. Phys.
Chem. Chem. Phys..

[24] Makepeace J. W., Hunter H. M. A., Wood T. J., Smith R. I., Murray C. A., David W. I. F. (2016). Ammonia decomposition catalysis using lithium–calcium
imide. Faraday Discuss..

