# Supplementary material for: Nature of the Lithium Amide–Imide Catalyst System under Ammonia Decomposition Conditions
Source: J Phys Chem C Nanomater Interfaces. 2025 Oct 3;129(41):18427–32. doi: 10.1021/acs.jpcc.5c04123 (PMC12536390; doi:10.1021/acs.jpcc.5c04123)
Supplement: Supplementary file 1 [file jp5c04123_si_001.pdf]

The Nature of the Lithium Amide-Imide Catalyst System under Ammonia Decomposition Conditions: Supplementary Information

Thomas J. Wood[†*] and Eleanor G. Frew[‡]

[†] ISIS Pulsed Neutron and Muon Source, Rutherford Appleton Laboratory, Harwell Oxford, Didcot, OX11 0QX, UK

[‡] Inorganic Chemistry Laboratory, University of Oxford, Oxford, OX1 3QR, UK

*Corresponding author: thomas.wood@stfc.ac.uk

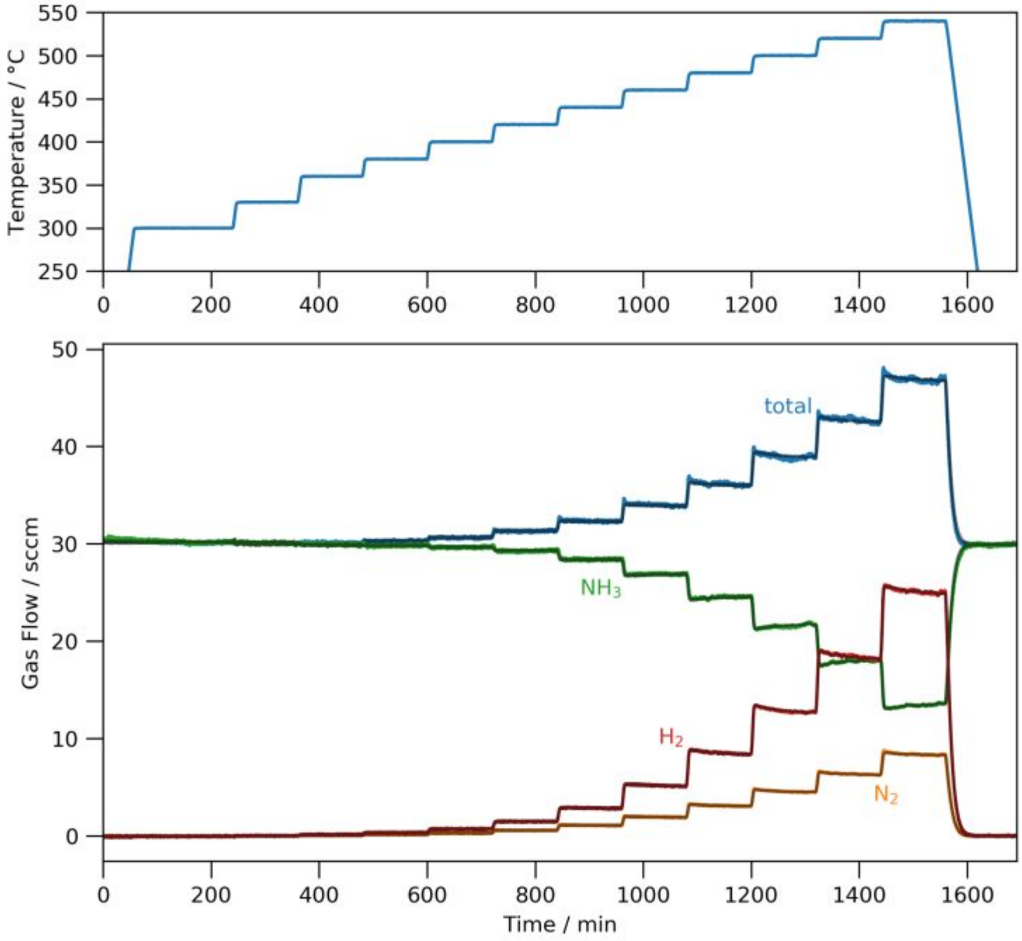

Figure S1: Gas component flows and temperature profile for the blank reactor; grey lines denote the expected flow from a system where only unreacted or decomposed ammonia comprises the outflow.

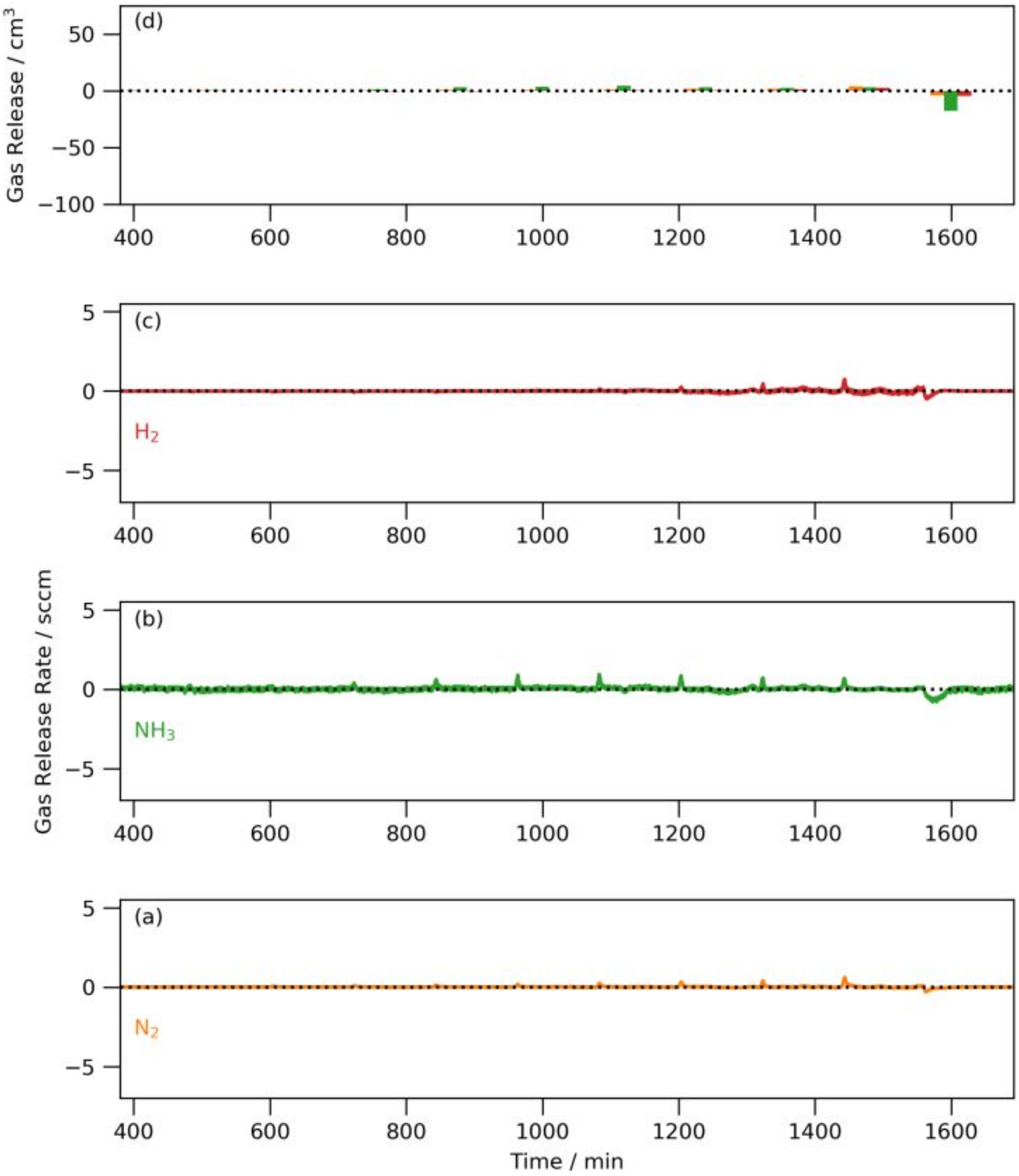

Figure S2: Flow due to gas released from walls of the blank reactor for (a) nitrogen; (b) ammonia; (c) hydrogen; (d) total volumes of gases released per temperature (bars offset from each other for clarity; orange = nitrogen, green = ammonia, red = hydrogen). The scale has been chosen to be consistent with Figure 6.

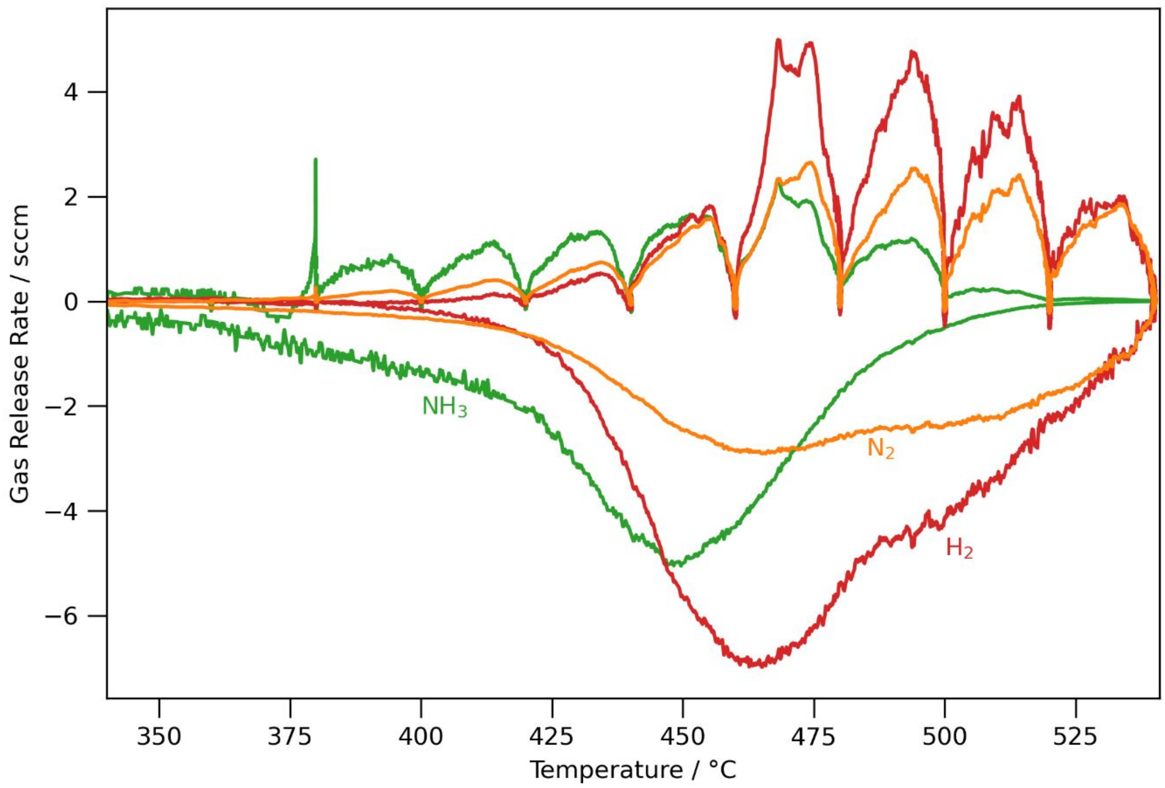

Figure S3: Gas release rates for both heating stages (positive releases) and cooling (absorptions) for the lithium amide catalyst.

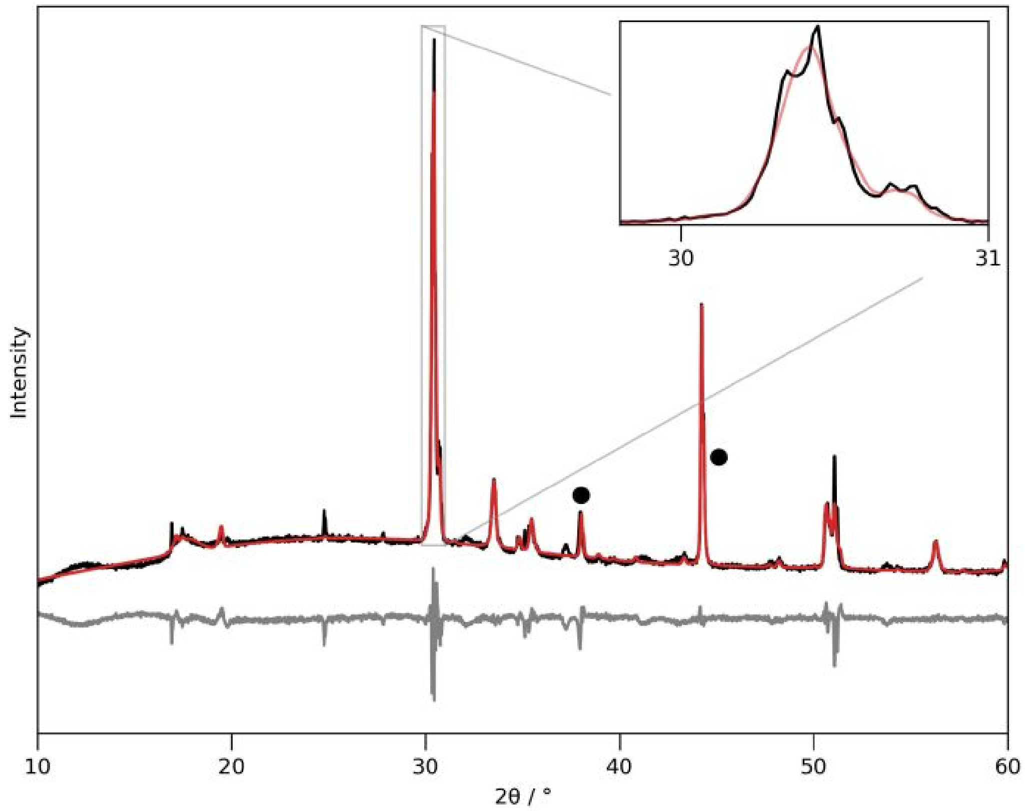

Figure S4: Powder X-ray diffraction pattern for lithium amide-imide catalyst post ammonia decomposition reaction; black line = collected data, red line = fit, r_{wp} = 5.6. The inset shows the 111 peaks of the imide-like phases and the 112 peaks of the amide-like phases. • refers to Al peaks from the sample holder.
